# “Navigating chaos”: Urban, Rural, and Remote Patient Experiences in Accessing Healthcare with Indigenous and Non-Indigenous Perspectives of Living with Chronic Low Back Pain

**DOI:** 10.1080/24740527.2024.2318706

**Published:** 2024-02-15

**Authors:** Katie Crockett, Stacey Lovo, Alison Irvine, Catherine Trask, Sarah Oosman, Veronica McKinney, Terrence McDonald, Nazmi Sari, Rosmary Martinez-Rueda, Harini Aiyer, Bertha Carnegie, Marie Custer, Stacey McIntosh, Brenna Bath

**Affiliations:** a School of Rehabilitation Science, University of Saskatchewan, Saskatoon, Saskatchewan, Canada; b Department of Biomedical Engineering and Health Systems, School of Engineering Sciences in Chemistry, Biotechnology, & Health, Royal Institute of Technology, Stockholm, Sweden; c Canadian Centre for Rural and Agricultural Health, University of Saskatchewan, Saskatoon, Saskatchewan, Canada; d College of Medicine, University of Saskatchewan, Saskatoon, Saskatchewan, Canada; e Department of Family Medicine and Community Health Sciences, University of Calgary, Calgary, Calgary, Alberta, Canada; f Department of Economics, University of Saskatchewan, Saskatoon, Saskatchewan, Canada; g Department of Community Health and Epidemiology, University of Saskatchewan, Saskatoon, Saskatchewan, Canada; h Patient Partner, University of Saskatchewan, Saskatoon, Saskatchewan, Canada

**Keywords:** Low back pain, healthcare access, health services, Indigenous, rehabilitation

## Abstract

**Background:**

Healthcare access for chronic low back pain is complex and should consider not only the health system, but patient care seeking experiences as well. People who live in rural and remote communities and/or identify as being Indigenous may often encounter additional barriers to accessing care for chronic low back pain; thus, these contexts must be considered to fully understand barriers and facilitators.

**Aims:**

The aim of this study was to understand care-seeking experiences of people living with chronic back pain in Saskatchewan and determine unique experiences facing urban, rural, remote, and/or Indigenous peoples.

**Methods:**

Thirty-three participants with chronic low back pain completed a preliminary survey followed by individual semistructured interviews. Participants were categorized as urban, rural, or remote including Indigenous status. A qualitative interpretive research approach with inductive thematic analysis was employed.

**Results:**

Three overarching themes were identified with the following subthemes: (1) healthcare access challenges: challenges to accessing care, challenges within the health system, and challenges leading to self-directed management/coping strategies; (2) healthcare access facilitators: funded care, participant education and knowledge, patient–provider communication, and care closer to home; and (3) participant recommendations for improved care provision: coordination of care, integrative and holistic care, and patient-centered care and support. Rural and remote participants highlighted travel as a main barrier. Indigenous participant experiences emphasized communication with healthcare providers and past experiences influencing desire to access care.

**Conclusion:**

Participants identified a range of challenges and facilitators as well as recommendations for improving access to care for chronic low back pain, with unique barriers for rural, remote, and Indigenous participants.

## RÉSUMÉ

**Contexte:** L'accès aux soins de santé pour la lombalgie chronique est complexe et devrait tenir compte non seulement du système de santé, mais aussi des expériences de recherche de soins des patients. Les personnes vivant dans des communautés rurales et éloignées et/ou qui s'identifient comme autochtones font souvent face à des obstacles supplémentaires pour accéder aux soins pour la lombalgie chronique; il faut donc tenir compte de ces contextes pour bien comprendre les obstacles et les facilitateurs.

**Objectifs:** L'objectif de cette étude était de comprendre les expériences de recherche de soins des personnes vivant avec une lombalgie chronique en Saskatchewan et de déterminer les expériences uniques d'accès aux soins auxquelles sont confrontées les personnes vivant en milieu urbain, rural, éloigné et/ou ayant un statut d’autochtone.

**Méthodes:** Trente-trois participants souffrant de lombalgie chronique ont répondu à un questionnaire préliminaire suivi d'entretiens individuels semi-structurés. Les participants ont été catégorisés comme vivant en milieu urbain, rural, éloigné, incluant ceux ayant un statut d'autochtone. Une approche de recherche qualitative interprétative avec une analyse thématique inductive a été utilisée.

**Résultats:** Trois thèmes principaux ont été répertoriés avec les sous-thèmes suivants : (1) difficultés d'accès aux soins de santé : difficultés pour accéder aux soins, difficultés au sein du système de santé et difficultés conduisant à des stratégies de gestion et d'adaptation autonomes; (2) facilitateurs de l'accès aux soins de santé : financement des soins, éducation et connaissances des participants, communication entre le patient et le prestataire de soins et proximité des soins par rapport au domicile et (3) recommandations des participants pour l'amélioration de la prestation des soins : la coordination des soins, les soins intégrés et holistiques, les soins et le soutien centrés sur le patient. Les participants des régions rurales et éloignées ont souligné que les déplacements constituaient un obstacle majeur. Les expériences des participants autochtones ont mis l'accent sur la communication avec les prestataires de soins de santé et les expériences passées qui influencent le désir d'accéder aux soins.

**Conclusion:** Les participants ont répertorié un ensemble de difficultés, de facilitateurs et de recommandations pour améliorer l'accès aux soins pour les lombalgies chroniques, qui présente des obstacles uniques pour les participants vivant en milieu rural et éloigné et les participants autochtones.

## Introduction

Healthcare access is a complex concept closely intertwined with health system performance.^1^ The most recent conceptual framework of access to healthcare presents a multidimensional view of healthcare access that factors in the capabilities of individuals and populations to perceive, seek, reach, pay, and engage in healthcare in addition to the dynamics between health systems and providers.^2^ This concept of access is important to consider given the known barriers to accessing healthcare in Saskatchewan, Canada.^3–5^ For Indigenous peoples in Canada, access to care must be contextualized by current health disparities as well as past and present individual and collective experiences of systemic racism and discrimination, including oppressive colonial systems that reinforce inequities in both care and health outcomes.^6–8^ Within the scope of this article, “Indigenous” refers to unique and distinctively different population groups, including First Nations (specifically Cree) and Métis Peoples.

Low back pain is a leading contributor of years lived with disability worldwide.^9^ Despite prevention efforts in Canada, the prevalence of chronic low back pain (CBP) remained stable between 2007 and 2014, with a higher prevalence among residents of rural locations.^10^ Saskatchewan is home to a high number of rural residents as well as Indigenous peoples; both groups are 30% more likely to experience CBP^11^ but are less likely to access care.^12^ It is well established that overall health outcomes are worse for Indigenous, rural, and remote residents compared to non-Indigenous and urban residents.^6,7,13,14^ Substantial gaps persist between evidence and practice for low back pain care^15^ that may be explained by contextual factors that shape both the health system and individual abilities. For example, the majority of Canadians seek care for CBP with a family physician^12^; however, it is estimated that over 4 million Canadians do not have a family doctor,^16^ with Canadian primary care moving into a crisis situation.^17^

Some barriers identified across Saskatchewan include cost/not having additional medical insurance, wait times, and geographic location.^4,5^ There are also known barriers unique to rural populations including high out-of-pocket expenses associated with having to travel long distances to urban centers to receive care and decreased access to clinicians who have specific knowledge in musculoskeletal management, including low back pain.^3,12^ Though potential barriers to healthcare access among Indigenous and non-Indigenous Canadians with CBP have been identified,^3–5,12^ no known study in Canada has used a mixed methods approach to understand the barriers and facilitators of healthcare access informed by the experiences of urban, rural, remote, and/or Indigenous peoples living with CBP.

This study aimed to (1) understand care-seeking experiences of people living with CBP in Saskatchewan and (2) determine the unique experiences (including additional barriers) facing urban, rural, remote, and/or Indigenous peoples.

## Methods

This article describes one phase of a multiphase study.^18^ The first phase involved one-on-one interviews with people with past or current CBP, as well as healthcare providers who serve them. The second phase included a province-wide telephone survey focused on access to healthcare barriers and facilitators among rural, remote, and urban people who have had or have CBP.^19^ The third phase consisted of a World Café method^20^ to facilitate interactive dialogue among people with lived experience of CBP, healthcare providers, health system policy, and decision makers. The protocol and methodology for the overarching study was previously published.^18^ For this phase, purposive sampling was used to recruit 33 participants (both Indigenous and non-Indigenous) from rural, remote, and urban Saskatchewan to understand diverse experiences with CBP. As such, data saturation may or may not have been attained. Recruitment attempted to proportionally reflect Saskatchewan’s geographical dispersion, with oversampling from rural areas due to the higher prevalence of CBP.^11,21^ Individuals 18 years or older, residing in Saskatchewan, and having a minimum of 3 months of current or historic low back pain were eligible to participate in the study. Low back pain was classified as pain between the 12th rib and gluteal fold with or without associated hip or leg symptoms. Geographic classification of rural, remote, and urban was determined by postal code and reference to the metropolitan influenced zone classification developed by Statistics Canada.^18^ Participants self-reported Indigenous identity and residence on reserve land.

Participants went through a screening and informed consent process. Participants received a paper or electronic consent form based on their preference. Consent was verified online prior to completing an online survey on sociodemographic and health information, baseline information on history and experience of low back pain, and types of services accessed (or not) for low back pain. Survey data were collected using REDCap (Research Electronic Data Capture), a secure online survey tool and research database application hosted by the University of Saskatchewan.^22,23^ Survey usability and technical functioning were tested by team members and patient partners beforehand.

Semistructured one-on-one interview questions were co-developed and tested with team members and patient partners to ensure clarity of questions and broad topic coverage, in line with a patient-oriented approach.^24^ Participants were asked about access to care from a multidimensional approach^2^ (Appendix A). Interviews were conducted virtually (Zoom or telephone) by one of the research team members (K.C. or A.I.) to explore participants’ experiences of living with back pain and accessing care, with consent verified again at the beginning and end of the interview. Interviews were audio-recorded and transcribed. Data were managed and analyzed using (NVivo 12.^25^ This project aimed to collect the lived experiences of healthcare access challenges and facilitators among and between people with CBP in rural, remote, and urban Saskatchewan and across Indigenous and non-Indigenous participants. We primarily collected the narratives and experiences of those living with CBP, and a qualitative approach provides the best insight to address this complex topic.^26,27^ Thematic analysis was conducted following Braun and Clarke’s approach.^27^ Analysis began with preliminary code development for the entire data set (R.M. and H.A.) and discussed and evaluated by periodic meetings by the research team (B.B., S.L., K.C., A.I., R.M., H.A.) to identify patterns and potential themes. Once the themes were identified, a check was carried out to identify adequate representation of the themes with quotes and the entire data set. Subsequent meetings were held with the research team, including patient partners, to further interpret and refine themes with select representative extracts. Trustworthiness was enhanced through an audit trail, as well as member-checking in the context of participant review of transcripts. Participants were provided their written transcripts and were able to make alterations to their data prior to data being analyzed. Reflexivity through continuous and collaborative work, as outlined above, further enhanced trustworthiness.

Inequities in the experience of chronic pain and accessing healthcare are intersectional in nature,^28^ and we recognize that positionality always has the potential to shape research processes. We feel that having a diverse team and specifically patient partners as full team members helped to provide a range of perspectives that were considered throughout the research process. The research team included multidisciplinary researchers, two primary care physicians, and three patient partners who represented different perspectives (urban, rural, and remote/Indigenous). To support reflexivity and an intersectional approach, the entire team met and collaborated on several occasions following a patient-oriented research approach. This also ensured that Indigenous worldviews, language, culture, community practices, and protocols informed how we conducted our research, including data analysis and manuscript preparation, in respectful, meaningful, and culturally relevant ways. Collectively, we have a team with extensive experience in community-based, Indigenous provision of and access to physiotherapy care in Saskatchewan and across Canada as well as population health and mixed methods research. We were particularly mindful of elevating Indigenous perspectives from Indigenous team members as well as from those who have a history of collaborative engagement with Indigenous communities and OCAP training.

This study was approved by the University of Saskatchewan Behavioral Research Ethics Board Beh-REB #1973.

## Results

Participants were from urban (*n* = 12, 36%), rural (*n* = 17, 52%), or remote (*n* = 4, 12%) areas of Saskatchewan. Seven participants identified as Indigenous (21%), with five residing on reserve land (15%). Participant ages covered a wide range (21–81 years), with dispersion across a range of education level and income. See Table 1 for participant demographics and treatment already accessed at the time of interviews.

**Table 1.** Participant demographics and treatment accessed.

Demographic factor	Urban (*n* = 12)	Rural (*n* = 17)	Remote (*n* = 4)	Total (*n* = 33)
Age (range and mean)	21–81 (52.8)	23–76 (54.9)	41–79 (67.5)	21–81 (56)
Sex	Female: 9	Female: 7	Female: 2	Female: 18
	(75%)	(41.2%)	(50%)	(55%)
	Male: 2	Male: 10	Male: 2	Male: 14
	(16.7%)	(58.8%)	(50%)	(42%)
	Other: 1 (8.3%)			Other: 1 (3%)
Indigenous (self-declared)	2 (16.7%)	4 (23.5%)	1 (25%)	7 (21%)
Residing on reserve land	0 (0%)	4 (23.5%)	1 (25%)	5 (15%)
Education				
Incomplete Grade 12	1 (8.3%)	2 (11.8%)	0 (0%)	3 (9%)
Trade school	3 (25%)	4 (23.5%)	1 (25%)	8 (24%)
Some university	3 (25%)	4 (23.5%)	2 (50%)	9 (27%)
University degree	4 (33.3%)	3 (17.6%)	1 (25%)	8 (24%)
Graduate degree	1 (8.3%)	3 (17.6%)	0 (0%)	4 (12%)
Income				
<$15,000	0 (0%)	2 (11.8%)	1 (25%)	3 (9%)
$15–29,999	2 (16.7%)	1 (5.9%)	1 (25%)	4 (12%)
$30–59,999	2 (16.7%)	7 (41.2%)	1 (25%)	10 (30%)
$60–99,999	5 (41.7%)	1 (5.9%)	1 (25%)	7 (21%)
>$100,000	3 (25%)	5 (29.4%)	0 (0%)	8 (24%)
Treatment already accessed				
Medication	9 (75%)	11 (64.7%)	3 (75%)	23 (70%)
Surgery	2 (16.7%)	2 (11.8%)	0 (0%)	4 (12%)
Physiotherapy	11 (91.7%)	12 (70.6%)	3 (75%)	26 (79%)
Chiropractic	7 (58.3%)	9 (52.9%)	2 (50%)	18 (55%)
Massage	5 (41.7%)	9 (52.9%)	3 (75%)	17 (52%)
Acupuncture	4 (33.3%)	6 (35.3%)	2 (50%)	12 (36%)
Exercise therapy	5 (41.7%)	6 (35.3%)	2 (50%)	13 (39%)
Traditional healing	1 (8.3%)	1 (5.9%)	1 (25%)	3 (9%)
Other^a^	2 (16.7%)	5 (29.4%)	1 (25%)	8 (24%)

^a^Gymnasium, walking track at community center, mental health counseling, specialist, pain clinic, yoga, osteopathy, edibles.

Representative quotes were selected from 4 of the 7 Indigenous participants and 16 of the 26 non-Indigenous participants. Of the 11 quotes used from the 4 Indigenous participants, the maximum number of quotes from any one participant is 4. Of the 27 quotes used from the 16 non-Indigenous participants, the maximum number of quotes from any one participant is 4.

## Interview Themes

**Figure 1.**
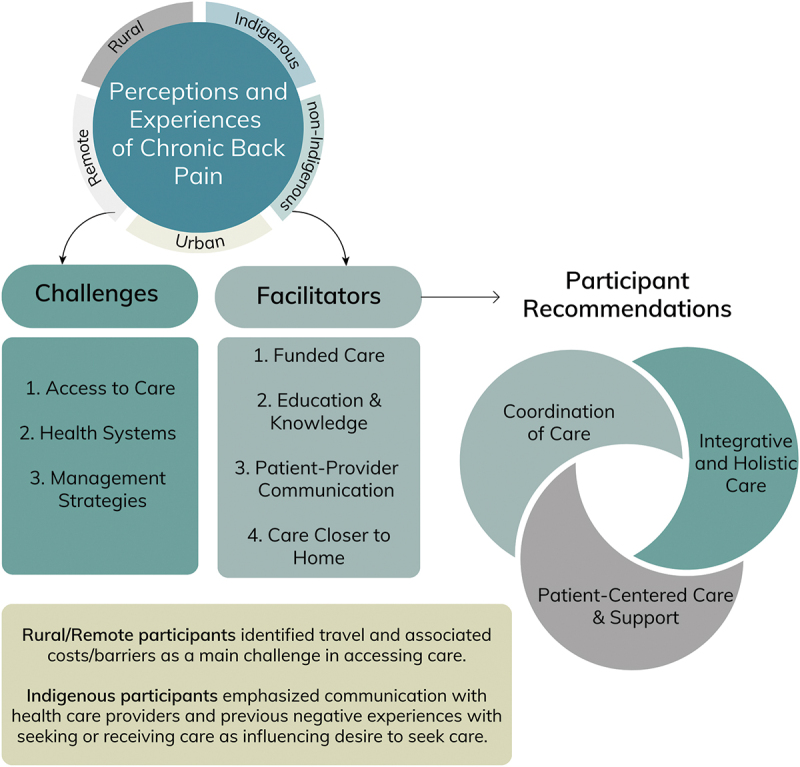
Graphic representation of themes.

There were three main overarching themes: (1) healthcare access challenges, (2) healthcare access facilitators, and (3) participant recommendations for improved care provision (Figure 1).

## Healthcare Access Challenges

To genuinely address the problems of healthcare access, various dimensions and complexities of healthcare access need to be considered from health system and patient perspectives. Challenges experienced by rural, remote, urban, and Indigenous participants were categorized into three areas: challenges to accessing care, challenges within the healthcare system, and challenges leading to patient coping strategies.

### Challenges to Accessing Care

Costs, wait times, extended travel times and distance, lack of available services, and perceived lack of training/knowledgeable healthcare providers were the most common perceived challenges.

Most services that non-insured health benefits cover are through the province. And if the province doesn’t cover it, then you’re out of luck. (Remote Indigenous participant)

I think having doctors that are more knowledgeable—like walk-in clinics, of course, are not really knowledgeable. So they can’t really help you as much as someone that specializes. But I know that’s unrealistic to have that. (Urban non-Indigenous participant)

Specifically for rural, remote, and Indigenous participants, there were additional costs to receive even publicly funded care if the services were not locally available (e.g., costs to travel: fuel, childcare, lost wages for patient and/or caregiver/travel companion).

Yeah, just the accessibility. If you don’t live in the big cities, then you—the traveling, like I said, that’s my biggest obstacle right now, is the traveling. (Rural Indigenous participant)

There was also a strong participant narrative of feeling like there is nothing that can be done about their CBP and that they need to “suffer” through their pain.

Well everybody’s told me there’s nothing you can do about [my back pain]. You just have to suffer. (Rural non-Indigenous participant)

Indigenous participants reported challenges in finding a family physician whom they trusted and with whom they felt safe.

Well, just their attitude. I don’t want to go [to the emergency department] unless if I’m dying, I’d go. (Rural Indigenous participant)

### Challenges with the Health System/Ongoing Challenges

Participant challenges with the healthcare system included system navigation, perceived lack of a diagnosis and management plan, not being believed, ineffective treatment, and lack of options for nonpharmacological care. Difficulties navigating the healthcare system and available options to provide help with their back pain were main challenges identified by participants. Participants reported the ongoing challenge and negative consequences of not having access to nonpharmacological care (e.g., side effects of medication, not getting better, quality of life not improving).

I guess part of the problem goes to, I don’t know, what is available for healthcare other than the emergency and our doctors. (Rural non-Indigenous participant)

Honestly, it would be nicer, I think, if the doctors were more open and upfront on the medication they’re giving you. ‘Cause they always give you a short—they go, “Don’t worry,” but for me it’s more I want to know about side effects also. I don’t want to be stuck taking a pill for the rest of my life that I shouldn’t be. (Rural Indigenous participant)

I don’t like pills. It was hard for me to find a doctor, I think, to listen to me, to try and explain that sometimes. (Rural Indigenous participant)

The lack of belief from healthcare providers invalidated patients’ lived experiences with pain and left them feeling frustrated and demoralized.

I was so nervous going into that appointment because—actually I’m always nervous meeting new doctors and anything medical right now—because of all those experiences of not being believed and being called a liar and [ma]lingerer and lazy and all those things, that I’m always very nervous when I see a new doctor. And so I walked in there with a lot of trepidation. (Urban non-Indigenous participant)

But the nurse there, she said to me, “I believe you,” and she was the first person to tell me that. And I was like, “Wow.” I was blown away when she said, “I can see that you’re in pain. I believe you.” And I know how much it hurts when people treat you like they don’t believe you. And I was like, “Wow, somebody’s telling me this?” … And when she told me that, I was like, “No physician, no nurse has told me this. Thank you.” (Remote Indigenous participant)

Indigenous participants emphasized previous negative experiences with seeking or receiving healthcare services influencing trust, desire, and feelings of safety when seeking care.

I think the trust, to me, is if people feel free and safe and welcomed, they will go, they will go to wherever they have to go and do what they’ve got to do. (Rural Indigenous participant)

Just what I said about having—not being judged when I walk in there, you know? That’s what I feel, so that’s been my experience. (Rural Indigenous participant)

### Challenges Leading to Self-Directed Management/Coping Strategies

Participants who could not or chose not to access care frequently reported coping behaviors, including fear avoidance strategies,^29^ due to a lack of self-management strategies or tools.

I’m very careful about what I do. We have grandchildren, so I’m extremely careful picking up and doing stuff and playing with them. I am more conscious about doing stretching and stuff, but I’m not really good about the keeping at it every day. I really haven’t had any direction, so it’s sorta kinda self-directed. Yeah, I have a lot of concern. I am very concerned. I do not want to go back to what I had. And so, I think there’s a little bit of—not fear, but a little bit of hesitancy about doing everything that I feel like I should be able to do. (Rural non-Indigenous participant)

I tried to do swimming and water walking, ‘cause that seemed a little bit better. But I think, generally, the fact that I had so much pain in a lot of sports and things is the reason I became kind of a couch potato. (Urban non-Indigenous participant)

Our one son told me, “Well you should be doing yoga.” And I tried looking online and I’m not sure what—I’m not even sure what would be a good starting point in doing that. And I don’t know what there is for a senior that has trouble bending. I don’t know what would be best or what would hurt too much. (Rural non-Indigenous participant)

The toll of chronic pain on mental health and the negative effects on participants’ ability to manage their back pain to a level of acceptable function was also a prominent theme.

I get madder now, I get frustrated ‘cause I can’t do nothing. So many things I’d like to do and can’t. So, I guess that’s emotional. (Remote non-Indigenous participant)

I was kind of down because it was taking longer than usual to resolve. And I thought, “Maybe this is going to be the new normal.” So it was kind of depressing. (Urban non-Indigenous participant)

## Healthcare Access Facilitators

When asked about factors that currently helped them access care for their CBP, participants cited both health system considerations and patient abilities,^30^ grouped into four subcategories: funded care, patient education, patient–provider communication, and care in the community.

### Funded care

Funded care identified by participants included both provincial Medical Services Plan coverage^29^ and extended health coverage through additional insurance (Workers’ Compensation and extended health benefits) and local community grants. Support that funded care provides is twofold: first, financial support with logistics related to access and, second, referral and navigation of the health system.

The fact that we don’t have to pay big money to see a specialist to me is very important, is something very, very positive. (Rural non-Indigenous participant)

Particularly for participants who were funded through systems like Workers’ Compensation, financial support for the process of accessing care (i.e., mileage) was covered.

When I was on WCB, then they would pay my mileage and all that stuff, which was really nice. But after that, it was a bit more eye-opening to see the extra costs. (Rural non-Indigenous participant)

Participants also acknowledged that funded care played a crucial role in their decision to seek services such as massage and physiotherapy.

I don’t know if I would’ve ever thought to go for massage if my healthcare didn’t help cover it. (Urban non-Indigenous participant)

### Participant Education and Knowledge

Education and knowledge were multifaceted and included understanding their pain, knowing what activities are safe, having a clear management plan, as well as ongoing support.

But the kinesiologist, that one thing that he said is, “No, you’re not harming yourself. It’s just your back telling you to be careful.” That was also a turning point. That also made a huge difference. A huge, huge, huge difference. (Rural non-Indigenous participant)

[The healthcare provider] shared her knowledge openly. Talked about each exercise. She explained it before and what it’s about, and she explained the area. So that way—okay, here is a good example—my neck and my side because of the sciatic nerve. She showed me some basic exercises so that pain in my neck—that it would help out the rest of my—how could I say that … I can do the exercise myself to heal myself and feel better. (Rural Indigenous participant)

Participants identified that educational components provided in one-on-one appointments helped them navigate care (knowing what services to access and having a management plan), facilitated self-management strategies, and reduced their fear of living with pain or causing further “harm.”

I found that if I pushed myself a little bit and I did those things, it actually helps, of course, as long as I’m not reckless or something. But if I’m careful about doing sports, or especially hiking and things like that, it does actually obviously really improve my quality of life and reduces the pain and everything. (Urban non-Indigenous participant)

I actually think I’m doing a pretty good job of managing it. I’ve gotten all the information over the years that I think I really need to succeed and be living a healthy life with my back pain. The only thing that I think would be helpful, maybe if there was more information online, readily available. (Urban non-Indigenous participant)

Having support groups (virtually or in-person) was identified by participants as an avenue to learn from peers and feel less alone.

I think having support from other people might be helpful … having support from other people that are experiencing the same thing is really important and helpful. (Rural non-Indigenous participant)

### Patient–Provider Communication

Relational continuity with a healthcare provider was a main facilitator identified by participants. Part of the trusting relationship included feeling heard and believed.

He listened to all my concerns, and you could tell that he was really paying attention … he was focused on me, he was in the moment, and that was—it was all-around just a very satisfying, happy, productive meeting. (Urban non-Indigenous participant)

For some participants, their healthcare providers were perceived as an advocate because they facilitated appropriate referrals, provided timely follow-up, and assisted with healthcare system navigation.

Before I do anything, I always consult with my family physician. He’s been my doctor since I was 15, I think. So, I start with him. And he’s really, really good about directing me to where I need to go or getting referrals to where I need to go (Urban Indigenous participant)

### Care Closer to Home

Participants who had care closer to or in their home community reported this as a facilitator to accessing care. This facilitator was more prominent with rural and remote participants, because they had the opportunity to compare circumstances to when care was not present in the community, with an understanding of the challenges to accessing care when travel is required.

The nurse practitioner was in [home community] at the community school, five days a week. And I had recently started seeing her when I switched my doctors to my physician in [another community], I just asked her. I said, “She’s in our community, would you mind if I went to see her, and would you work with her if I have to go see her for whatever?” And my doctor said, “Sure, no problem.” Yeah, that was very helpful. (Rural non-Indigenous participant)

Here on the reserve, for hands-on healing, people can just go to the hands-on—my sister. They can go see my sister at where she’s practicing, and there’s no questions asked. They get covered. I think the Indian Band itself covers them, not through non-insured health benefits, like, not through the province. (Remote Indigenous participant)

Urban participants reported having care closer to home as a facilitator as well but with less importance. Additionally, improved access to nonpharmacological management was emphasized as a facilitator to improving pain management, including negative side effects of medication use.

I think that people, no matter where they live in the province, should have direct access to the minimum—a GP. A GP that understands them that can direct them to the appropriate physio or whatever care that they need. (Urban non-Indigenous participant)

## Participant Recommendations for Improved Care Provision

Participants recommended improvements in three specific areas: coordination of care, establishing more integrative and holistic care, and a focus on patient agency and patient-centered care.

### Coordination of Care

At a structural level, participants emphasized the need for enhanced coordination of care between providers and patients, in conjunction with broader health system–wide coordination, to enhance access to and provision of care for back pain. Participants reported that having providers who are more knowledgeable regarding chronic pain management, including knowledge on what resources are available to their patients with back pain, would be helpful.

Having my doctors communicate with each other a little bit more would be helpful. (Rural Indigenous participant)

Having some doctor or team of doctors that would—not even just doctors, but professionals—that would be able to analyze or figure out what’s going on and then give you some recommendations and tell me what I need to be doing. That would really help. (Rural non-Indigenous participant)

Team-based care or continuity of care to ensure a cohesive management plan was mentioned as placing less burden on or overwhelming patients in explaining, re-explaining, and feeling alone in managing their condition or deciphering conflicting messages from various practitioners.

To see the same doctor, like time after time after time is difficult because there’s such a rotation of them. But it just seems like you repeat the story and start all over again, every time you see a new doctor. (Remote non-Indigenous participant)

### Integrative and Holistic Care

Participants emphasized the need to transition from a narrow view of localized back pain to a holistic and integrative approach, with the benefit of long-term improvement in their health.

I had a physio once [who] so well explained the pathway of how pain affects the nerve responders and affects how you perceive the pain and how your mental health can all be affected […], just having that approach, to be able to explain it in, like, “Yes, yes this is all affected. It’s not your fault that you’re having these—it’s not your fault that your mental health is affected, it’s just that it’s all related, and we need to kinda work on you as a whole person.” (Rural non-Indigenous participant)

Holistic care was described as a whole-body approach that incorporates different modalities of care.

A holistic approach, too, to have all those different aspects of care. ‘Cause, I mean, you can’t just fix one thing and be on the way, but looking at the whole person, a more holistic approach, should definitely be more patient-centrred and more, probably, beneficial for everyone. (Rural non-Indigenous participant)

Integrative care suggestions included exercise classes, exercise support groups, counseling, yoga, and mindfulness.

It would be great if there were counselors that had that understanding and that could help us get better, ‘cause … a big part of chronic pain is how you feel about it. And how you’re treated. And if you’re not treated well and you end up with more pain because of that, then you’re not making any progress. (Urban non-Indigenous participant)

### Patient-Centered Care and Support

Participants expressed a desire to receive more information on their back pain and care options to make informed, collaborative decisions.

I wanna be more involved in my decision making, as well. I just don’t wanna be told what I’m supposed to do, like it just seems like I wanna be part of the solution, too, not just kind of left in the dark, I guess. (Remote Indigenous participant)

When participants were provided with individualized self-help strategies, they reported benefits both physically and mentally.

I think I want to say that just, like, the self-help strategies that chiropractor provided me with to empower me to make better decisions on how to attack dealing with back pain—I think mindfulness, self-talk, those kind of things, probably, in my mind, they seem to be the most impactful. (Rural Indigenous participant)

Definitely getting some stretches to do, getting some exercises, getting some advice. And especially even just mentally, the idea that I was doing something about it, that was very helpful to me. (Rural non-Indigenous participant)

Participants reported the benefit of supportive practitioners through encouragement and identifying positive changes in function despite ongoing pain.

And she helped with that. and she was very encouraging, and she was observing me without me knowing. And then she would say, “I’ve noticed that you’re not leaning your elbows on the table or everything anymore to support yourself. That’s progress.” And yeah, you’re right! She’s just such—so positive and not like she was watching me until I screwed up or anything like that, it was all very subtle and it was all the positives that she would pick up on. And then challenge me to do a little bit more until next time. That was very positive. (Urban non-Indigenous participant)

## Discussion

Overlapping themes were present among participant-identified challenges and facilitators across participant groups. Findings revealed gaps in access to care from both the health system and practitioner, as well as patients’ abilities to access care.

A significant proportion of Canadians are without a family physician, who serve as gatekeepers to specialist care and other health services.^16,31^ Furthermore, the dominant fee-for-service model that many Canadian physicians operate under often does not allow adequate time for patient education and comprehensive assessment, particularly for complex healthcare challenges, such as CBP.^32^ After ruling out specific spinal pathology, primary care guidelines for nonspecific low back pain recommend offering high-quality education that supports patients’ return to activity, encouraging exercise, and self-management/self-care, which family physicians may support but do not have the time, resources, skills, and knowledge to adequately implement as a sole practitioner.^33–35^ Participants who reported experiences with these facilitating elements reported improvement with their back pain and greater ability to manage and live with their condition.

Many participants reported years of frustration in trying to get pain relief, with the perception that psychosocial factors were not addressed or acknowledged, limiting a return to the pre-pain lifestyle. Perceptions of unmet needs for people accessing care for CBP have been previously reported, attributed to misconceptions and mismatch of patient expectations to clinical guidelines.^32,36^ In this study, both Indigenous and non-Indigenous participants across all levels of rurality reported a lack of trust and feeling that their provider did not believe them, demonstrating the previously established stigma for those who seek care for chronic pain.^37^ Medication use as a primary treatment option was commonly cited by participants alongside multifaceted barriers to nonpharmacological care. Many participants reported feeling that medical management alone was ineffective, in addition to unpleasant side effects. This is especially concerning given the current opioid crisis in Canada^38^ and the understanding of overmedicalization of CBP and that excessive medical investigations and low-value healthcare approaches can increase the risk of long-term back-related disability.^39^ Current guidelines for CBP recommend taking into account the patient’s expectations and preferences,^34^ with emphasis on shared decision-making principles.^33^

Health system navigation and knowledge of accessible services and resources was reported as both a challenge and recommendation. For example, access to physiotherapy in Saskatchewan does not require a physician referral for self-paying individuals, but a referral is needed if it is delivered through the provincial health system, Medical Services Branch (MSB). Many participants did not know this. There was additional confusion and complexity for Indigenous participants and navigating the Non-Insured Health Benefits process, which has been previously reported as a challenge.^40^ When relying on Non-Insured Health Benefits, Indigenous peoples are limited in provider choice and location of services, often with considerable time and travel requirements, including navigating transportation barriers and medical taxi services. Across groups, participants reported waiting on guidance from their family physician for referrals or care-seeking recommendations or were under the impression that “nothing could be done.” Other research on chronic pain management in Saskatchewan resulted in patient-identified key areas for improvement, including care navigation and coordination, education, and supporting engagement and fostering wellness, among others.^41^

Participants broadly perceived lack of specific knowledge for CBP management among primary care providers, which may be related to contextual and systematic limitations; the current system does not allow adequate time for assessment of highly complex issues, including low back pain/chronic pain, to be fully addressed.^32^ Participants suggested that team-based care could help “figure out what’s going on.” Team-based care was reported in various contexts of multi- or interdisciplinary care with all types of providers within the patient’s circle of care being in communication with each other to offer consistent/complementary management plans but also work collaboratively to address potential underlying causes. Team-based or coordinated care that would allow for assessment and treatment that included and emphasized education on CBP may be more reflective of current needs that would meet both patient and practitioner needs. Team-based care may improve care while reducing burden on physicians and allow efficient access to musculoskeletal providers and appropriate chronic pain education.^17,32^ However, in rural and remote areas where local services are not available, future models of care should consider lack of local services when making recommendations and how to support sole care providers in rural and remote areas. Virtual team models of care for CBP may be a potential solution to further explore.^42–44^ Given that cost was a main challenge in accessing care, it is appropriate that more coordinated approaches for improvements include funding for mental health and physical therapies, incentivizing this high-value care.

The findings of this study demonstrate overlapping themes among Indigenous and non-Indigenous participants; however, there were unique experiences among Indigenous participants. Previous negative experiences with healthcare services or providers among Indigenous peoples in Canada have repeatedly been found to pose a barrier to accessing healthcare. Experiences of racism and discrimination contribute to a lack of trust in providers and the system, ultimately resulting in refusal to seek or delay in seeking care.^45,46^ The impact of historical and/or intergenerational trauma influences Indigenous peoples’ overall health and well-being, including healthcare–seeking behavior.^6,7,14^ This, combined with the internal stigma of chronic pain, leads to anticipation of devaluation and discrimination and further avoidance coping behaviors, affecting access to care.^37^ Geographic location added a barrier of having to travel, with compounding barriers leading to reduced desire to access care in some cases.

### Future Recommendations

Access to healthcare includes enabling people to enter in contact and obtain appropriate healthcare, with the right to reach or visit a healthcare provider and/or use healthcare services.^2^ Future research should investigate how to disseminate knowledge and resources to individuals living with CBP, including, but not limited to, treatment options consistent with current guidelines, available appropriate services, and pharmaceutical and nonpharmacological management options. From our patient-focused interviews, there is evidence to support system changes to facilitate informed decision making among patients as well as providers and provide financial support for evidence-based nonpharmacological treatment.^32^

### Strengths and Limitations

This study has several limitations. Patient experiences presented in this article may be community specific and therefore not applicable broadly. Though specific interview questions related to the COVID-19 pandemic were asked and participants were asked to speak about their experiences broadly, these interviews were conducted during the height of public health restrictions; thus, experiences shared may be shaped by this context. There were several mitigative strengths of this project, including using a patient-oriented research approach and integrating experiences related to COVID-19 changes in provision of healthcare.

### Conclusion

Study participants perceived a lack of education and guidance to manage CBP, a lack of accessible and/or appropriate services, and challenges navigating health systems as restricting their ability to seek and receive care. Additionally, prior experiences influenced the desire to seek care. Though there are several shared experiences among Saskatchewan residents living with CBP, unique geographical factors (rural/remote/urban) and the experiences of Indigenous peoples with systemic racism ultimately determine individual experiences. There is a pressing and urgent need for CBP care models to address the barriers identified in this article to mitigate inequities in access to CBP care in regions with high proportions of rural and/or Indigenous peoples.

## Appendix A: Interview Guide

**Preamble:**

Hello, my name is X, and I am part of the research team looking at how easy or difficult it is for people with back pain to get the help and care they need. Thank you for agreeing to talk to me about your experiences with having low back problems.

As you know from the consent form we just reviewed together, the information you share today will help us to better understand your personal experiences with back pain (both positive and negative) and ways we might be able to measure and understand future changes to accessing healthcare in your community.

The information you share will help us to better understand what care you need for your back pain at the time that you need it, which may also be helpful to other people in a similar situation.

Your information will be combined with information shared by others from across Saskatchewan who have also experienced low back pain, as well as healthcare providers for those with low back pain (like doctors, nurses, or physiotherapists, for example). We plan to share our findings with policy and decision makers, with the hope of improving access to healthcare services for you and other residents of Saskatchewan.

We want to acknowledge the environment that we are in today with COVID-19. Your experience today may be very different from your initial experiences in accessing healthcare for your back pain. During our interview, try to think of your long-term experiences and the big picture and not just what is going on today. We know that the last few months will have influenced your experience, and we would like to hear about your full experience, so hopefully our questions will allow you to share your full experience.

This interview/discussion should take approximately 30 minutes to 1 hour. As we reviewed in the consent form, I will be recording our conversation today and the recording will then be typed up into a written script for analysis after the research project is completed. Are you okay with proceeding with the interview at this time?

Do you have any questions before we get started?

*If no …*

Let’s get started. I am turning on the recorder now.

1. Tell me a bit about your back pain. *(pause after this statement and wait for a response; if needed, use the following bulleted list as probes to draw out information from the participant)*

2. Can you tell me about when your back pain started?

 (a) Has it been constant since it started, or has it come and gone?

3. How has having back pain affected your life?

 Prompts:

  (a) Physical abilities?

  (b) Social participation? That is, activities with family, friends, community, work.

  (c) Emotional consequences?

  (d) Your ability to practice your cultural and/or spiritual activities?

  (e) Other?

  (f) Has this changed since COVID-19?

4. What types of supports/services have you tried for your back pain? What has been helpful?

 Prompts:

  (a) Healthcare services? (including physical therapy services? Medications?)

  (b) Community supports?

  (c) Others? (e.g., companion animals)

  (d) Local/traditional cultural practices?

5. Can you tell me about any challenges you have had in trying to access healthcare for your back problems?

 Prompts:

  (a) Travel?

  (b) Wait times?

  (c) Financial/ costs?

   (i) Need for accommodation while away? Food and/or per diem? Food reimbursement adequate for your needs?

   (ii) Ability to pay for a companion to join you for your appointments?

  (d) Cultural?

  (e) Comfort interacting with healthcare professionals?

  (f) Other?

(Additional prompt considering services responses above):

*Which services were impacted by these challenges?*

*Has this changed since COVID-19?*

6. What types of other services and/or supports do you think would help to support you in better treating and managing your low back pain and overall abilities to do the things you want to do?

7. Can you tell me about things that have helped you get the care you need for your back problems?

 Prompts:

  (a) For example, something that helped remove financial barriers, geographic/location barriers, wait times, or cultural mismatch?

  (b) Extra health insurance?

  (c) Publicly funded service?

  (d) Care in the community?

  (e) Quick access to healthcare providers?

  (f) Others?

8. Can you tell me about a time when you got care for your back problems and it turned out well?

 Prompts:

  (a) What was good about it?

  (b) What about that experience made it helpful?

  (c) Anything that could have improved it even more?

  (d) Are there any other experiences you can share where you had a positive experience?

9. Can you tell me about a time when you got care for your back problems and it did NOT turn out well?

 Prompts:

  (a) What was negative about it?

  (b) What about that experience made it negative?

  (c) Anything that could have improved your experience?

  (d) Are there any other experiences you can share where you had a negative experience?

10. What would help make it easier for you to access healthcare?

11. What are things that are meaningful or important to you when it comes to improving access to care for your back problems?

 Prompts (use terms in brackets):

  (a) Affordability (costs, travel time, how you are able to pay for services)

  (b) Availability and accessibility (location of services, hours of opening, appointment mechanisms, short wait times)

  (c) Appropriateness (quality of care, trusting relationship, effectiveness of care)

  (d) Acceptability (professional values/beliefs are in line with your own, cultural awareness/understanding/connection, gender)

  (e) Approachability (transparency, services that make themselves known and use language that is understandable)

12. If changes in healthcare services occurred in your community, do you have any suggestions on how we could measure whether these changes were successful in improving care?

*Pause and use the following prompts if needed*:

 (a) The experiences/stories of people with back pain?

 (b) Less pain? Measuring pain levels?

 (c) Better quality of life? (clarifying what types of activities you are now able to participate in, that you could not do before receiving appropriate healthcare)

 (d) Better movement/mobility?

 (e) More able to participate in social/community activities?

 (f) Less use of prescription medicines?

 (g) Less travel from the community?

 (h) Others?

13. Over the past several months, healthcare practices have been forced to change due to COVID-19. How have these changes impacted your ability to access the care that you have needed for your back?

 Prompts:

  (a) Have you been able to continue receiving care?

  (b) Has the way in which you have received care changed? How?

  (c) What has been difficult about these changes?

  (d) What has been easier for you in accessing healthcare providers? (i.e., appointments being held over the phone or videoconference)

14. Is there anything else you would like to share with us about either your experiences with back problems, healthcare access, or use of healthcare services?

 (a) (Once they stop talking) anything else we should know?

Thanks so much for your time and your thoughts. Before we finish today, I would like to go back to the consent form briefly. Now that we have been through the interview and you know what you have shared with me, I just want to go back through the sections we checked off and see whether you still consent in the same way as before we started. It is perfectly okay to change your mind on any of this. [At this point, confirm all the check box decisions with the participant.]
